# Stent-graft delivery across a highly tortuous proper hepatic artery using balloon anchoring, an inner dilator, and partial unsheathing

**DOI:** 10.1016/j.radcr.2026.05.079

**Published:** 2026-06-23

**Authors:** Jun Matsuda, Masahiro Kurozumi, Shun Imai, Megumi Fuke, Takateru Yamamoto, Marika Shimizu, Tetsuta Nishikawa, Takeshi Suzuki

**Affiliations:** a Department of Radiology, Nagano Municipal Hospital, 1333-1 Tomitake, Nagano 381-8551, Japan; b Department of Cardiovascular Surgery, Nagano Municipal Hospital, 1333-1 Tomitake, Nagano 381-8551, Japan; c Department of Radiology, Shinshu University School of Medicine, 3-1-1 Asahi, Matsumoto, Nagano 390-8621, Japan

**Keywords:** Hepatic artery aneurysm, Stent graft, Vessel tortuosity, Balloon-anchoring technique, Guiding sheath, Partial unsheathing technique

## Abstract

Proper hepatic artery aneurysms are a subset of visceral artery aneurysms. Due to the risk of rupture, treatment is recommended for aneurysms of the proper hepatic artery measuring ≥ 20 mm in diameter. To preserve hepatic blood flow, treatment consisting of autologous graft interposition, vascular bypass surgery, or stent-graft placement is recommended for such aneurysms. Here, we report the case of a man in his 70s with concurrent aneurysms of the proper hepatic artery, right gastric artery, and right gastroepiploic artery. He had severe pulmonary emphysema, which precluded general anesthesia, making him ineligible for surgical intervention. Therefore, we planned coil embolization of the right gastric and right gastroepiploic artery aneurysms and stent-graft placement for the proper hepatic artery aneurysm. Vessel tortuosity was marked, with a tortuosity index of 1.67, which was higher than that previously reported; therefore, stent-graft deployment was anticipated to be extremely challenging. In highly tortuous vessels, vessel straightening can produce an accordion-like appearance with step-like deformities, thereby impeding stent-graft advancement. Using a balloon-anchoring technique and an inner dilator, we successfully advanced the guiding sheath to a position distal to the aneurysm, achieving successful placement of the stent-graft. There were no periprocedural complications, and the stent-graft remained patent at the 3-month follow-up. Antiplatelet therapy is ongoing. These techniques may represent a viable approach for stent-graft insertion into highly tortuous vessels.

## Introduction

Visceral artery aneurysms, including those of the proper hepatic artery, are rare, and hepatic artery aneurysms account for 20% of all visceral artery aneurysms [1]. They are classified as either true aneurysms or pseudoaneurysms. True aneurysms are associated with etiologies such as atherosclerosis and genetic or autoimmune disease, whereas pseudoaneurysms may arise from trauma, mycotic aneurysms, or iatrogenic causes [1,2]. Most are asymptomatic, but they may present with abdominal pain or, in the case of hepatic artery aneurysms, hematemesis or melena secondary to hemobilia. Rupture can be fatal. In this paper, we report the successful placement of a stent-graft in an anatomically challenging case of a proper hepatic artery aneurysm with concomitant right gastroepiploic and right gastric artery aneurysms in a patient who was ineligible for surgical intervention.

## Case report

A man in his 70s was found to have a proper hepatic artery aneurysm on a physical checkup and consulted his previous physician. Progressive enlargement of the aneurysm was observed over time, and treatment was deemed necessary. Due to severe pulmonary emphysema, respiratory rehabilitation was attempted but did not improve his condition sufficiently to permit general anesthesia, making surgical intervention infeasible. The patient was therefore referred to our hospital for endovascular treatment.

Contrast-enhanced computed tomography (CT) revealed a 26 × 23 × 29 mm saccular aneurysm at the bifurcation of the common and proper hepatic arteries. This represented a 5 mm increase compared with the measurement obtained 3 years earlier. The course from the common hepatic artery to the proper hepatic artery was markedly tortuous, with an efferent-to-afferent angle of 15°. The vascular length between the splenic artery bifurcation and the left hepatic artery bifurcation was 55 mm, and the vessel diameter was 5 mm. Additional small aneurysms were identified in the right gastric artery and right gastroepiploic artery (Fig. 1).

**Fig. 1:**
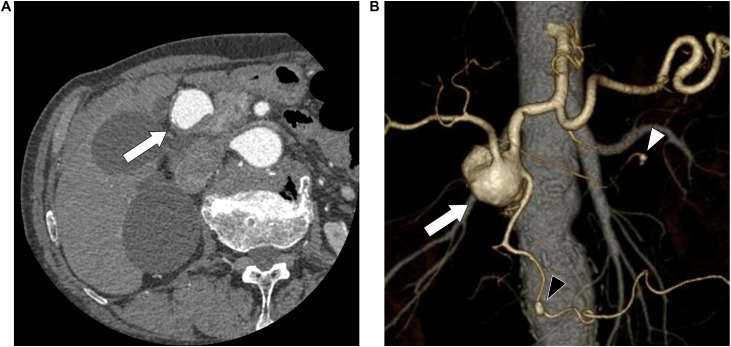
Preoperative contrast-enhanced CT. (A) Axial image. (B) Volume-rendered image. A 26 × 23 × 29 mm saccular aneurysm at the bifurcation of the common hepatic artery and the proper hepatic artery (arrow). The course from the afferent to the efferent artery is highly tortuous. Additional aneurysms are observed in the right gastric artery (white arrowhead) and right gastroepiploic artery (black arrowhead).

We considered the appropriate treatment strategy as follows (Fig. 2): embolization of the proper hepatic artery aneurysm would render subsequent treatment of the right gastric and right gastroepiploic artery aneurysms in the distal branches impossible. Conversely, if the right gastric and right gastroepiploic artery aneurysms were embolized first, occlusion of the proper hepatic artery aneurysm would disrupt collateral circulation, thereby increasing the risk of hepatic ischemia. For these reasons, we elected to perform the treatment in 2 stages: Initial coil embolization of the right gastric and right gastroepiploic artery aneurysms, followed by stent-graft placement for the proper hepatic artery aneurysm (Fig 3).

**Fig. 2:**
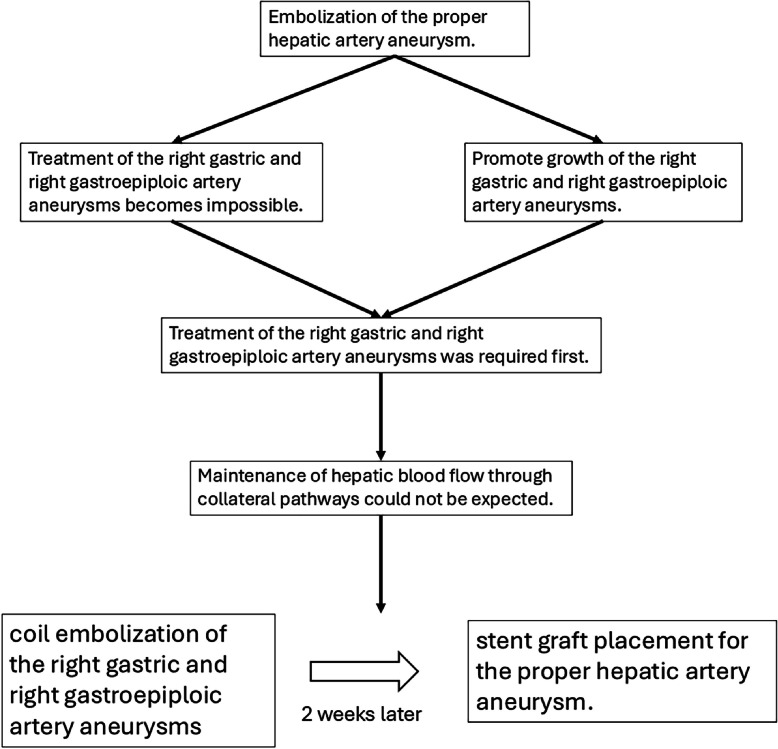
Determination process of treatment strategy.

**Fig. 3:**
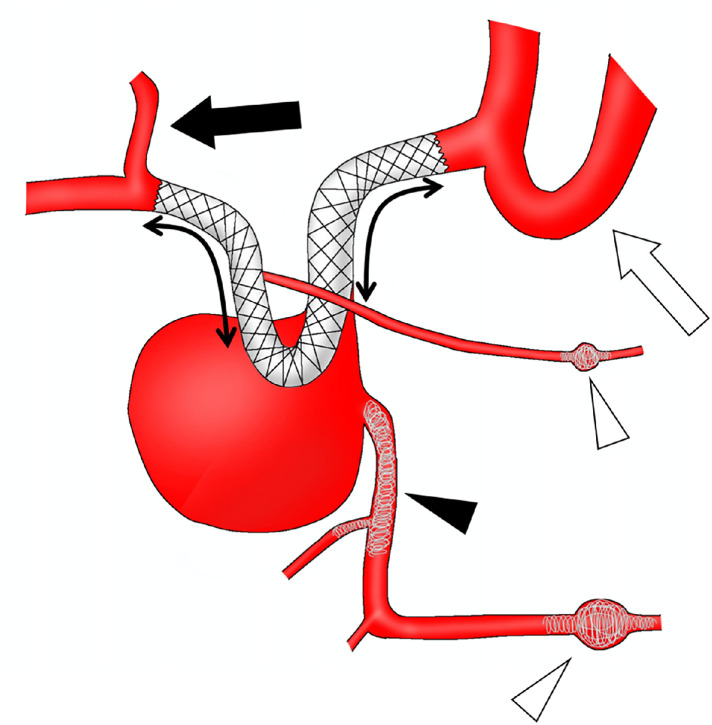
Schematic overview of the treatment strategy. First, the right gastric artery aneurysm and the right gastroepiploic artery aneurysm (white arrowhead) are coil-embolized, followed by coil embolization of the gastroduodenal artery (black arrowhead) to prevent endoleaks. Subsequently, a stent-graft is deployed between the splenic artery bifurcation (white arrow) and the left hepatic artery bifurcation (black arrow). Based on CT measurements, the landing zones (between the arrows) were estimated to be 13 mm both proximally and distally.

***Treatment of the right gastric and right gastroepiploic artery aneurysms (***Fig. 4***):*** Under local anesthesia, the right femoral artery was punctured, and a 5.5-Fr sheath introducer was inserted. A 5-Fr guiding catheter (Medikit Guiding Catheter HOK Shepherd Hook; Medikit, Tokyo, Japan) was positioned in the celiac artery. An attempt was made to advance a 4-Fr distal access catheter (DAC) (Cerulean G; Medikit) into the gastroduodenal artery over a 0.035-inch hydrophilic guidewire (Radifocus; Terumo, Tokyo, Japan); however, this was not possible because the celiac artery branched caudally from the aorta at a steep angle. Therefore, using a 2.9-Fr microballoon catheter (Logos Grandmaster; Piolax, Kanagawa, Japan) and a 0.014-inch micro-guidewire (Begin; Asahi Intecc, Aichi, Japan), the right gastroepiploic artery was catheterized, and the DAC was advanced into the gastroduodenal artery using a balloon-anchoring technique. A 1.9-Fr microcatheter (Carnelian Marvel; Tokai Medical, Gifu, Japan) threaded through a 2.9-Fr steering catheter (Leonis Mova; SB-Kawasumi, Kanagawa, Japan) was advanced to a position distal to the right gastroepiploic artery aneurysm. The aneurysm and the parent vessel proximal to it were embolized using one detachable coil (Target XL 360 Soft; Stryker, Kalamazoo, MI, USA; 3 mm/9 cm × 1) and 5 pushable coils (C-stopper 14; Piolax; 10 cm × 4, 3 cm × 1). To prevent endoleaks during subsequent stent-graft placement for the proper hepatic artery aneurysm, a 2.9/1.9-Fr microcatheter (Breakthrough; Boston Scientific, Marlborough, MA, USA) was inserted, and the gastroduodenal artery was embolized using 3 detachable coils (Target XL 360 Soft; Stryker; 5 mm/10 cm × 1; Target XXL 360; Stryker; 6 mm/20 cm × 2) and 3 pushable coils (C-stopper 16; Piolax; 10 cm × 2, 6 cm × 1). Next, the 2.9-Fr steering catheter and 1.9-Fr microcatheter were advanced into the right gastric artery, and the 1.9-Fr microcatheter was positioned distal to the right gastric-artery aneurysm. Embolization was performed using one detachable coil (Target Tetra; Stryker; 2.5 mm/6 cm × 1) and 6 pushable coils (C-stopper 14; Piolax; 10 cm × 6). To confirm whether hepatic blood flow would be preserved in the event of common-to-proper hepatic artery occlusion during the subsequent procedure, a 3.8-Fr microballoon catheter (Pinnacle Blue; Tokai Medical) was inserted into the common hepatic artery, and angiography of the celiac artery was performed with the common hepatic artery occluded. Digital subtraction angiography (DSA) showed no hepatic blood flow via collateral pathways. Portal venous flow was preserved. The fluoro time was 51 min, radiation dose was 463 mGy, and contrast volume was 102 mL (Iopamidol-300; Bayer, Nordrhein-Westfalen, Germany).

**Fig. 4:**
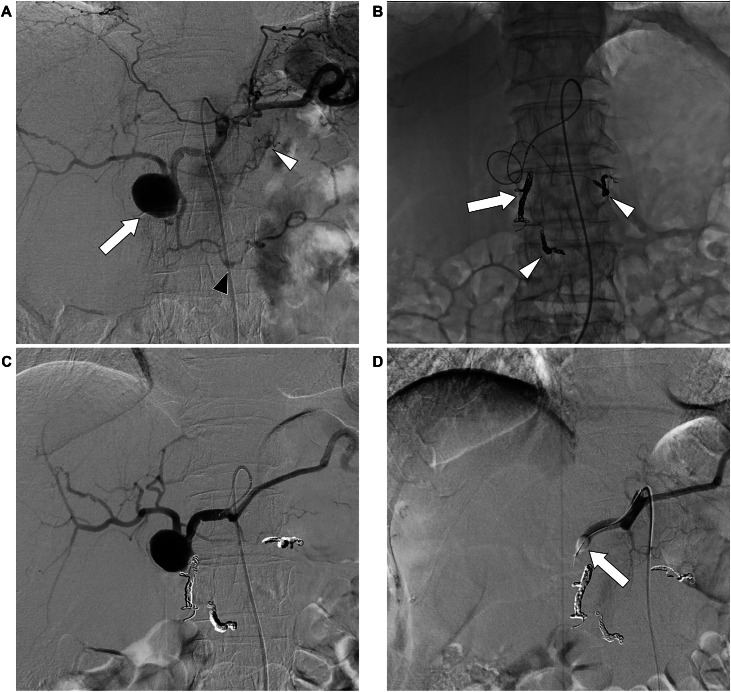
Treatment of the right gastric and right gastroepiploic artery aneurysms. (A) Digital subtraction angiogram (DSA) of the celiac artery showing the proper hepatic artery aneurysm (arrow), right gastric artery aneurysm (white arrowhead), and right gastroepiploic artery aneurysm (black arrowhead). (B) The right gastric and right gastroepiploic artery aneurysms are embolized with coils (arrowhead), and the gastroduodenal artery is embolized with a coil (arrow). (C) Post-embolization DSA of the celiac artery showing cessation of blood flow in the embolized vessels. (D) DSA of the celiac artery with balloon occlusion (arrow) of the common hepatic artery. No collateral blood flow to the liver is seen.

***Treatment of the proper hepatic artery aneurysm (***Fig. 5***):*** The proper hepatic artery aneurysm was treated 2 weeks after the procedure for the right gastric and right gastroepiploic artery aneurysms. For thromboprophylaxis, 3000 units of heparin were administered, followed by 1000 units every hour throughout the procedure. Because the celiac artery branched caudally from the aorta at a steep angle, a left brachial artery approach was selected to ensure reliable delivery of the stent-graft. Under local anesthesia, the left brachial artery was punctured, and a 6-Fr guiding sheath (Destination; Terumo) was inserted. For intra-aneurysmal access and confirmatory angiography during stent-graft deployment, the right femoral artery was punctured under local anesthesia, a 5.5-Fr sheath introducer was inserted, and a 4-Fr catheter (Glidecath II; Terumo) was positioned in the celiac artery. The 6-Fr guiding sheath was advanced into the common hepatic artery, and a 4-Fr DAC (Cerulean G; Medikit) and a 2.9-Fr microballoon catheter (Logos Grandmaster; Piolax) were inserted, with the microballoon catheter advanced to the A8 branch of the right hepatic artery. Using a balloon-anchoring technique, the DAC was advanced to the A8 branch of the right hepatic artery, eliminating catheter meandering within the aneurysm (Fig. 5A, B). Distal advancement of the guiding sheath was intended to facilitate controlled partial unsheathing during stent deployment. A 0.014-inch supportive micro-guidewire (Chevalier; Nipro, Osaka, Japan) was inserted through the microballoon catheter, and an attempt was made to advance the guiding sheath to a position distal to the aneurysm using the balloon-anchoring technique; however, this was unsuccessful. The balloon-anchoring technique combined with the 0.014-inch support wire did not provide sufficient backup force to advance the guiding sheath through the tortuous segment within the aneurysm. After removing the microballoon catheter, a 0.035-inch spring guidewire (Amplatz Extra-Stiff Wire; Cook Medical, Bloomington, IN, USA) was inserted through the DAC, and a further attempt was made to advance the guiding sheath; this too was unsuccessful. Step-like deformities of the vessel were observed on DSA. We hypothesized that these deformities produced by vessel straightening were the obstacle. The DAC was therefore switched for the inner dilator of the guiding sheath using a catheter exchange technique. Then, by advancing the guiding sheath very slowly while carefully confirming that the patient was not experiencing pain, it was advanced to a position distal to the aneurysm (Fig. 5C). The vascular length was measured during withdrawal of the spring guidewire, and the distance between the splenic artery bifurcation and the left hepatic artery bifurcation was approximately 6 cm. Accordingly, a 6 mm diameter × 5 cm self-expanding stent-graft (Viabahn; W.L. Gore & Associates, Newark, USA) was selected. The anticipated landing zone length was approximately 13 mm at both the proximal and distal ends (Fig. 3). With reference to the DSA (Fig. 5C), the distal end of the stent-graft was positioned just proximal to the bifurcation of the left hepatic artery. A 0.018-inch stiff wire (V-18; Boston Scientific) was inserted, and the stent-graft was advanced along the guidewire. As a countermeasure against endoleaks following stent deployment, a 2.7-Fr microcatheter (Carnelian HF-S; Tokai Medical) was inserted into the aneurysm through the 4-Fr catheter via the right femoral artery approach. To mitigate the bowstring phenomenon, the stent-graft was deployed while progressively unsheathing the guiding sheath (Fig. 5D, E). After confirming the absence of endoleaks and that the landing zone was separated from the splenic artery bifurcation and the left hepatic artery bifurcation on angiography, the intra-aneurysmal microcatheter was removed (Fig. 5F). Post-dilation was not performed because of the concern that balloon expansion could cause dislocation of the stent-graft at the tortuous segment and because no endoleak was observed. The fluoro time was 62 min, radiation dose was 619 mGy, and contrast volume was 191 mL (Iopamidol-300; Bayer).

**Fig. 5:**
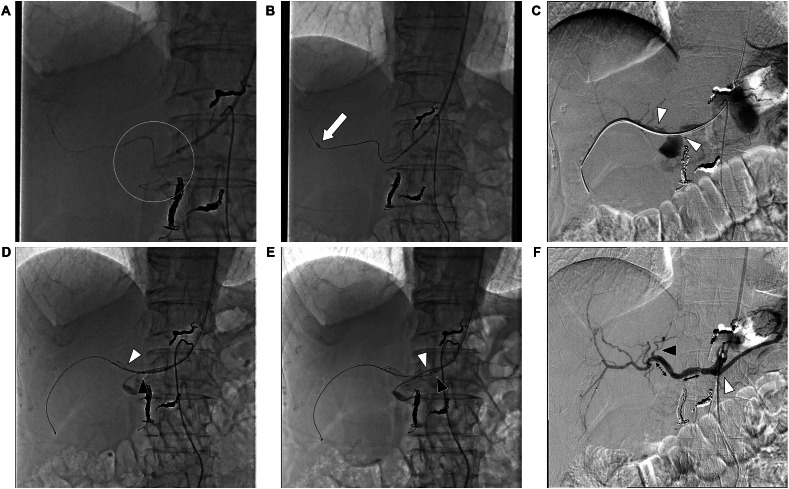
Treatment of the proper hepatic artery aneurysm. (A, B) Meandering of the catheter within the aneurysm (white circle) was eliminated using a balloon-anchoring technique (arrow). (C) DSA after advancement of the guiding sheath to a position distal to the aneurysm. The vessel is straightened, and accordion-like appearances and steps (arrowheads) were observed. (D, E) The stent-graft is deployed using a partial unsheathing technique. The stent-graft (white arrowhead) is deployed while progressively withdrawing the guiding sheath (black arrowhead). (F) Post-deployment DSA demonstrating preservation of hepatic blood flow with no findings suggestive of endoleaks. The proximal landing zone (dashed line) and distal landing zone (solid line) were each separated from the splenic artery bifurcation (white arrowhead) and the left hepatic artery bifurcation (black arrowhead), respectively.

On postoperative day 1, heparin was administered as a continuous intravenous infusion at 10,000 units/d. From postoperative day 2, oral dual antiplatelet therapy with aspirin 100 mg/d and clopidogrel 75 mg/d was initiated. No changes in liver function were observed in blood examinations conducted before and after treatment. At the 1-month follow-up, the stent-graft lumen was patent with no findings suggestive of migration or endoleaks (Fig. 6). At the 3-month follow-up, no changes were observed, and magnetic resonance angiography is scheduled for the subsequent 1-year follow-up. Dual antiplatelet therapy is ongoing.

**Fig. 6:**
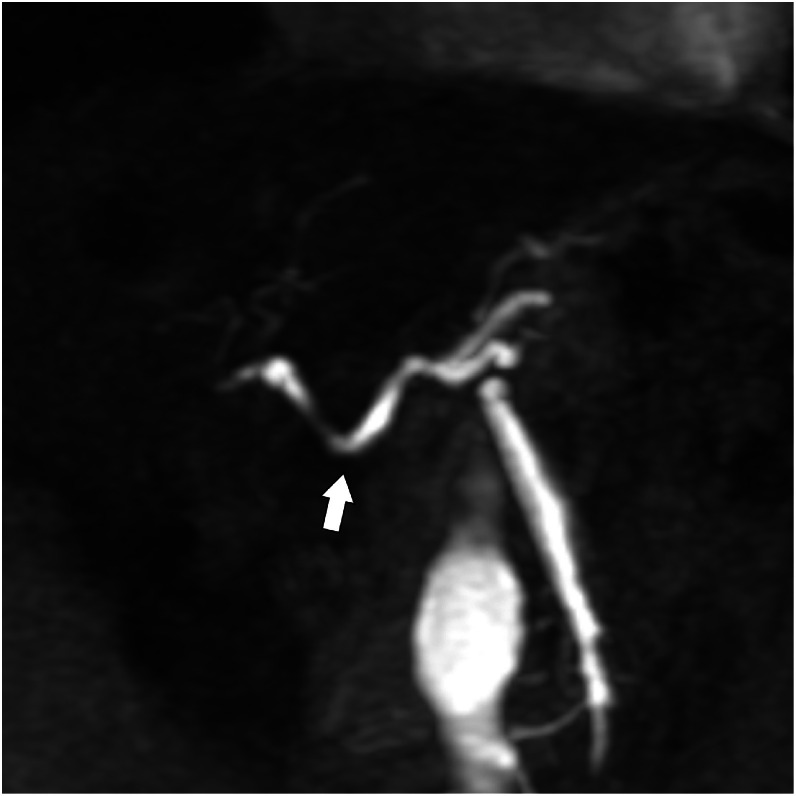
Contrast-enhanced MRI at 1 month postoperatively. Coronal contrast-enhanced T1-weighted imaging confirmed stent patency (arrow) with preservation of hepatic blood flow. No findings suggestive of migration or endoleaks are observed.

## Discussion

In this case, the proper hepatic artery aneurysm measured 29 mm and demonstrated progressive enlargement over time. These findings met the treatment indication criteria outlined in several guidelines, which recommend intervention for aneurysms measuring ≥ 20 mm [3,4]. Because visceral artery aneurysms are rare, consensus has not yet been reached regarding the optimal treatment. However, autologous graft interposition, vascular bypass surgery, or stent-graft placement are recommended by several authors to preserve hepatic blood flow in the treatment of proper hepatic artery aneurysms [1,3]. In the present case, general anesthesia was precluded by pulmonary emphysema, and endovascular treatment was the only viable option. Studies comparing coil embolization and stent-graft placement for hepatic artery aneurysms have reported that stent-graft placement is preferable in terms of preventing hepatic ischemia [5]. Furthermore, in this patient, aneurysms were present in the right gastric artery and right gastroepiploic artery, and embolization of the proper hepatic artery could have promoted growth of these additional aneurysms. Treatment of these aneurysms was required first, and maintenance of hepatic blood flow through collateral pathways could not be expected. For these reasons, we adopted a stent-graft strategy.

Hassold et al. [6] reported that the patency rate after stent-graft placement was 84% at 30 days, decreasing to 42% at 1 year. Although clear evidence regarding the relationship between antiplatelet therapy and stent-graft occlusion remains lacking, dual antiplatelet therapy has been empirically administered in many reported cases. In addition, some reports have described stent-graft occlusion following discontinuation of clopidogrel [7], suggesting that continuation of antiplatelet therapy may be important for maintaining stent-graft patency.

In situations where the celiac artery branches caudally from the aorta at a steep angle, insertion of the guiding sheath is more readily accomplished via a brachial artery approach [8]; hence, a left brachial artery approach was selected in our case.

In cases of marked arterial tortuosity, self-expanding stent-grafts achieve higher deployment success rates than balloon-expandable stent-grafts [9]. We used the Viabahn, a self-expanding stent-graft, which is well-suited for tortuous vessels owing to its high flexibility [5]. The Viabahn is deployed in a distal-to-proximal direction by pulling and unraveling a circumferential suture. In highly torturous lesions, excessive tension on the distal end of the system during deployment can cause it to assume a spherical configuration (the bowstring phenomenon) [10], resulting in deployment failure or migration. To prevent the bowstring phenomenon and facilitate stent delivery, a technique has been described in which the guiding sheath is advanced to a position distal to the aneurysm, and the stent-graft is deployed while the sheath is progressively withdrawn using the partial unsheathing technique [8,11]. This technique was employed in the present case. To conform to the tortuous vessel and reduce the risk of vascular injury, a Destination guiding sheath with a flexible tip was used.

It has been reported that greater vessel tortuosity is associated with increased difficulty in stent-graft deployment [1,9]. In tortuous vessels, coil embolization may also be considered because of the potential presence of collateral circulation [7]. Although the present case involved more severe vessel tortuosity than previously reported cases, stent-graft placement was attempted because collateral circulation could not be expected, as described above.

Okumura et al. [12] reported that, in cases of highly tortuous vessels, straightening of the vessel produces accordion-like appearances accompanied by step-like deformities, impeding advancement of the stent-graft (Fig. 7A). When a guiding sheath cannot be advanced within a straightened vessel, step-like deformities observed on DSA may represent a potential obstacle. The vessel tortuosity index in the present case, calculated by tracing the arterial path from the celiac artery to the destination point on DSA images and dividing it by the straight-line distance between these 2 points (Fig. 8), was 1.67, which was higher than values reported in prior studies. In the present case, accordion-like appearances and steps were observed upon vessel straightening. Okumura et al. [12] managed this by switching to a softer guidewire; however, this approach may not permit delivery of the stent-graft and does not eliminate the risk of the bowstring phenomenon. We successfully advanced the guiding sheath past the steps to a position distal to the aneurysm and deployed the stent-graft using a partial unsheathing technique. Factors contributing to the success of this approach included the use of the balloon-anchoring technique [13] to advance the DAC to a peripheral position and straighten the catheter course within the aneurysm, as well as insertion of a stiff guidewire through the DAC. Together with the inner dilator and guiding sheath, this enabled the sheath to be advanced past the accordion-like appearance and steps (Fig. 7B).

**Fig. 7:**
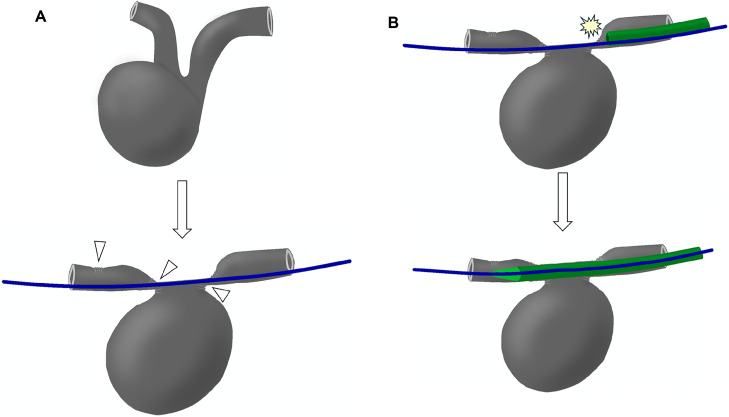
Schematic representation of angiographic findings during vessel straightening. (A) Straightening of a highly tortuous vessel produces accordion-like appearances and steps (arrowheads). (B) Use of the inner dilator of the guiding sheath enables the catheter to be advanced past the narrowed step segments.

**Fig. 8:**
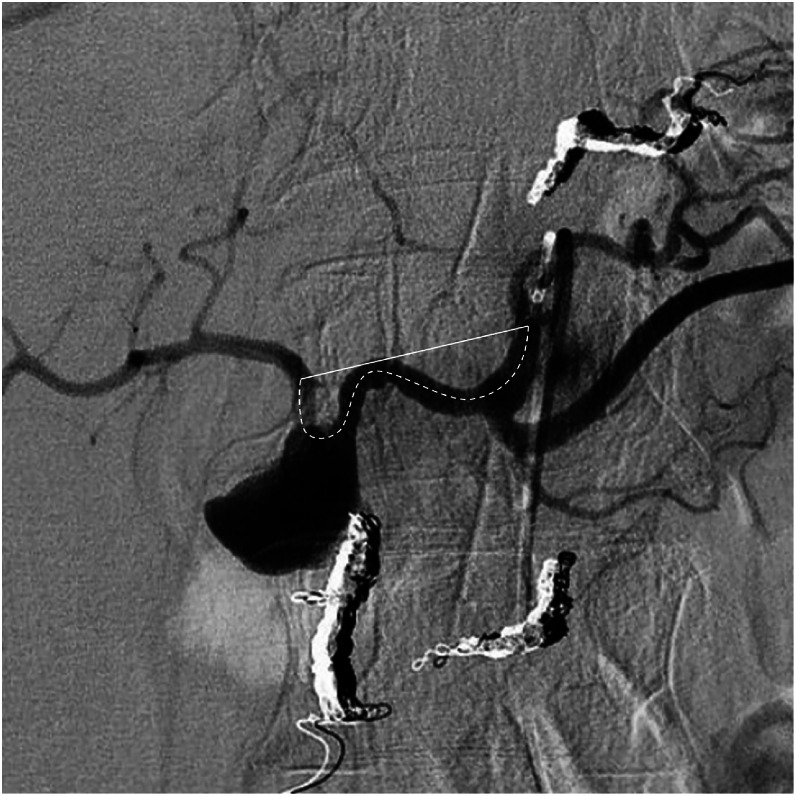
Vessel tortuosity index was calculated by tracing the arterial path from the celiac artery to the destination point on DSA images (white dashed line) and dividing it by the straight-line distance between these 2 points (white solid line).

The technical success rate of stent-graft placement is very high, at 92%-100%, with few periprocedural complications [5,11,12]. However, a potential risk of this method is that advancing a dilator through a vessel under substantial mechanical stress may cause hemorrhage or dissection. We advanced the guiding sheath gradually over an extended period and ultimately achieved successful insertion. However, it is essential to ensure the guiding sheath has a smaller caliber than the target vessel and to advance it under frequent angiographic confirmation while carefully verifying the absence of patient pain and hypotension. In addition, as balloon expansion of a tortuous stent-graft may cause dislocation, the decision to perform post-dilation should be made with caution.

In conclusion, stent-graft placement was performed for a proper hepatic artery aneurysm in a patient ineligible for surgical intervention. Stent-graft deployment in highly tortuous vessels is challenging. Use of a balloon-anchoring technique and an inner dilator to advance the guiding sheath past accordion-like appearances and steps may enable stent-graft placement in highly tortuous vessels.

A limitation of this study is the short follow-up period of 3 months; thus, the long-term prognosis remains unknown.

## Patient consent

Written informed consent was obtained from the patient to publish their clinical and imaging data.
